# Modeling the Flowering Activation Motif during Vernalization in Legumes: A Case Study of *M. trancatula*

**DOI:** 10.3390/life14010026

**Published:** 2023-12-23

**Authors:** Maria A. Duk, Vitaly V. Gursky, Maria G. Samsonova, Svetlana Yu. Surkova

**Affiliations:** 1 Mathematical Biology and Bioinformatics Laboratory, Peter the Great Saint Petersburg Polytechnic University, 195251 St. Petersburg, Russia; 2 Theoretical Department, Ioffe Institute, 194021 St. Petersburg, Russia

**Keywords:** vernalization, legumes, gene networks, dynamical model, feed-forward loop, *Medicago trancatula*, *FT*, *SOC1*, *PIM*

## Abstract

In many plant species, flowering is promoted by the cold treatment or vernalization. The mechanism of vernalization-induced flowering has been extensively studied in *Arabidopsis* but remains largely unknown in legumes. The orthologs of the *FLC* gene, a major regulator of vernalization response in *Arabidopsis*, are absent or non-functional in the vernalization-sensitive legume species. Nevertheless, the legume integrator genes *FT* and *SOC1* are involved in the transition of the vernalization signal to meristem identity genes, including *PIM* (*AP1* ortholog). However, the regulatory contribution of these genes to *PIM* activation in legumes remains elusive. Here, we presented the theoretical and data-driven analyses of a feed-forward regulatory motif that includes a vernalization-responsive *FT* gene and several *SOC1* genes, which independently activate *PIM* and thereby mediate floral transition. Our theoretical model showed that the multiple regulatory branches in this regulatory motif facilitated the elimination of no-sense signals and amplified useful signals from the upstream regulator. We further developed and analyzed four data-driven models of *PIM* activation in *Medicago trancatula* in vernalized and non-vernalized conditions in wild-type and *fta1-1* mutants. The model with *FTa1* providing both direct activation and indirect activation via three intermediate activators, *SOC1a*, *SOC1b*, and *SOC1c*, resulted in the most relevant *PIM* dynamics. In this model, the difference between regulatory inputs of *SOC1* genes was nonessential. As a result, in the *M. trancatula* model, the cumulative action of *SOC1a*, *SOC1b*, and *SOC1c* was favored. Overall, in this study, we first presented the in silico analysis of vernalization-induced flowering in legumes. The considered vernalization network motif can be supplemented with additional regulatory branches as new experimental data become available.

## 1. Introduction

In many plant species, flowering is induced by the cold treatment or vernalization [1,2]. Vernalization inhibits precocious reproductive development during winter and ensures that flowering occurs in the milder spring conditions [3].

**Figure 1:**
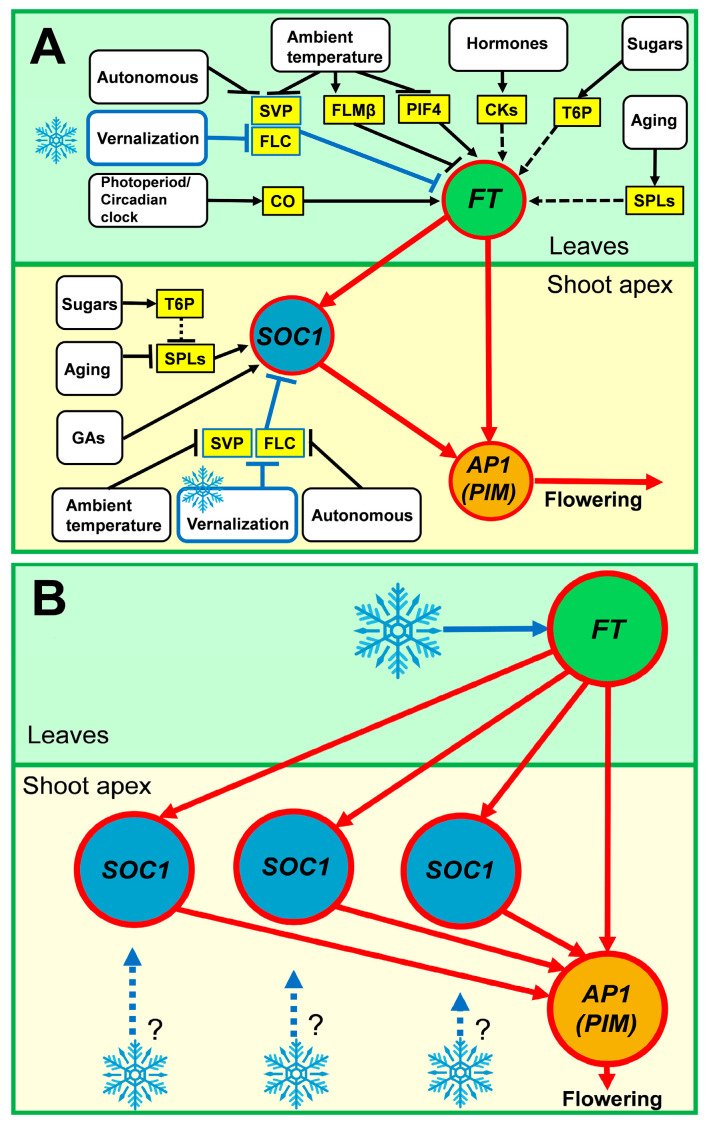
A core regulatory motif responsible for flowering activation by vernalization in (**A**) *Arabidopsis* and (**B**) legumes. (**A**) In *Arabidopsis*, the *FT* and *SOC1* genes integrate regulatory inputs from multiple signaling pathways. In the non-inductive conditions, these integrators are repressed by the *FLC* gene. Cold treatment leads to *FLC* repression and activation of *FT* and *SOC1*, which in turn activate the meristem identity genes, including *AP1 (PIM)*. This turns on floral transition. *FT* activates meristem identity genes both directly and via *SOC1*. The scheme is based on the pathway overview from the Flor-ID database (http://www.phytosystems.ulg.ac.be/florid/, accessed on 4 December 2023) and a previously published summary on mechanisms of floral transition in *Arabidopsis* [19,20]. Arrows and T-bars show positive and negative regulatory interactions, respectively. Dashed lines correspond to indirect/putative mechanisms. GAs, Gibberellins; CKs, cytokinins. (**B**) In legumes, the mechanisms of vernalization-induced flowering have not yet been sufficiently studied. Each legume species has several *FT* genes, but often only one *FT* gene is involved in the vernalization response. Upon cold treatment, the *FT* gene activates *SOC1* genes, which are present in two or three copies of the legume genomes. It is largely unknown whether the vernalization signal transduction from *FT* to the *AP1 (PIM)* is direct or indirect and whether the legume *SOC1* genes perceive any vernalization signal directly. The blue snowflakes and arrows denote the vernalization signaling pathway, whose components are well characterized in *Arabidopsis* but remain unknown in legumes.

The mechanisms of vernalization response are best studied in *Arabidopsis* [4]. In *Arabidopsis*, the *FLOWERING LOCUS T (FT)* and *SUPPRESSOR OF OVEREXPRESSION OF CONSTANS 1 (SOC1)* genes are the major hubs collecting regulatory inputs from many signaling pathways (Photoperiod/circadian clock, Vernalization, Ambient temperature, Gibberellins/cytokinins, Autonomous, Sugar, and Aging) to transmit this information to the meristem identity genes, including *APETALA1 (AP1)*, a master regulator of flower development [5,6,7,8,9,10] (Figure 1A). The major regulator of the vernalization pathway is the *FLOWERING LOCUS C (FLC)* gene [3,11,12,13], while the information on photoperiod and circadian clock is integrated by the *CONSTANS (CO)* regulator [14]. In the non-inductive conditions, FLC represses *FT* and *SOC1* integrator genes by binding to the first intron of *FT* and the promoter region of *SOC1* in a complex with SHORT VEGETATIVE PHASE (SVP) [9,15,16,17]. The vernalization treatment switches an epigenetic mechanism of *FLC* silencing, which leads to the de-repression of the *FT* and *SOC1* [6]. Upon de-repression, the *FT* gene becomes activated in leaves by the CO protein, which binds to the cis-element in the *FT* promoter [18]. Then, the FT protein moves to the shoot apex to activate the expression of the meristem identity genes and promote floral transition [7] (Figure 1A). A mechanism of the vernalization-induced silencing of the floral repressor is generally conserved in cereals [4].

In legumes, the molecular bases of vernalization response are still largely unexplored [21]. The available data suggest significant differences in the mechanisms of vernalization response between legumes and *Arabidopsis.* The *FLC* orthologs are missing in many vernalization-sensitive legume species, including *Medicago truncatula*, *Cicer arietinum*, *Pisum sativum*, and *Lupinus angustifolius* [22,23]. An overexpression of *SVP* genes in transgenic *M. trancatula* did not alter flowering time [24]. Moreover, the studies in *M. truncatula* and *P. sativum* suggested that *COL* genes, the legume orthologs of the *Arabidopsis CO* gene, may not be involved in the regulation of photoperiodic flowering [25,26].

Despite differences in response to vernalization and photoperiod, the function of integrator and meristem identity genes is generally conserved in legumes. The legume *FT* genes are proposed to be the main targets of vernalization [21,22,27]. Unlike *Arabidopsis*, temperate legumes have four to six *FT* genes arranged in three subclades, but not all of these genes are involved in the vernalization response [27]. For example, the *FTa1* gene has been reported to be a vernalization target in *M. trancatula* and *P. sativum* [28,29,30], while the *FTc1* gene plays a major role in vernalization response of *L. angustifolius* and *L. luteus* [31,32]. In *C. arietinum*, the candidate vernalization targets have not yet been reported.

The *SOC1* genes are usually present in two or three copies in the legume genomes, and their expression is vernalization-sensitive [33,34]. However, it is unclear whether they perceive the vernalization signal directly or indirectly and whether their function is redundant (Figure 1B). For meristem identity genes, the number of known orthologs varies between legume species; however, one of the major genes involved in the floral transition is the *PROLIFERATING INFLOWERING MERISTEM (PIM)*, an ortholog of the *Arabidopsis AP1* gene [35,36,37].

During the floral transition, the *FT* and *SOC1* genes function as pathway integrators [5,6], and given the absence of detailed information on their upstream regulators in legumes, these genes can be considered inputs of the regulatory network. According to this concept, the core regulatory motif promoting vernalization in legumes should include an *FT* gene and two or three *SOC1s*, which regulate the target meristem identity genes (Figure 1B). It is still unknown how vernalization triggers flowering in legumes or how the positive environmental signals propagate within this network motif. We studied this motif using mathematical modeling based on both theoretical assumptions and gene expression data.

Since the studies on vernalization-induced flowering in legumes are only beginning to unfold, the data on expression dynamics of the integrator genes during vernalization are available only for *M. trancatula*. In *M. trancatula*, the *FTa1* gene is the main target of vernalization and the long day (LD) photoperiod. The *fta1* mutants lose the vernalization response [28]. In addition, *M. trancatula* has three *SOC1* genes (*SOC1a*, *SOC1b*, and *SOC1c*), whose expression is up-regulated by vernalization [33,38]. These genes are suggested to be downstream of *FTa1* as their expression declines significantly in the *fta1* mutants. Nevertheless, the vernalized *fta1* mutant plants still flower in the LD conditions, although with a delay. This is accompanied by an increase in *SOC1s* expression [38]. Each *SOC1* gene may have a specific function during floral transition. In *M. trancatula*, the mutations in the *SOC1a* gene led to delayed flowering in all examined conditions, while the mutation in *SOC1b* did not affect flowering time [38]. The data on *SOC1c* mutants are not yet available [33,38]. Thus, many questions remain unanswered. It is unclear whether the vernalization signal transmission from the vernalization-responsive *FTa1* gene to the shoot apex requires cumulative input from the *SOC1* genes or whether each *SOC1* gene has its own role. If the flowering still occurs in the *fta1* mutants, do the *SOC1* genes perceive any vernalization signal independently of *FTa1*?

Mathematical modeling is an effective tool to analyze interactions within gene networks. Various kinds of models have been applied to infer the mechanisms of floral transition in *Arabidopsis* [39,40,41,42]. Models based on ordinary differential equations (ODEs) mostly considered a core gene network that included an activator *FT* and a repressor *TERMINAL FLOWER1 (TFL1)*, both mediated by the *FD* gene, as well as their target meristem identity genes *AP1 (PIM)* and *LFY* [40,41]. It has been shown that the floral transition networks include the major hubs, which can be expanded by additional hubs and interactions [40]. In line with these assumptions, a more sophisticated model, including *SOC1* and *AGAMOUS-LIKE24 (AGL24)* regulators, has been elaborated [43]. Recently, the ODE models were successfully applied to analyze the networks of floral transition in legumes. The interactions between the *FT*, *FD*, *TFL1*, *LFY*, and *AP1 (PIM)* orthologs were modeled in *C. arietinum* [44] and *P. sativum* [45]. However, no mathematical models of vernalization-induced flowering in legumes have yet been developed.

**Figure 2:**
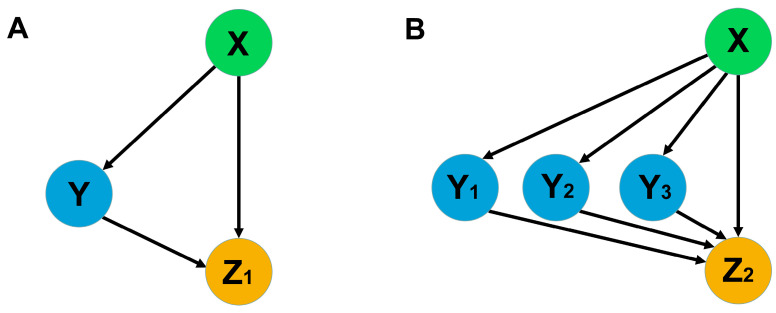
The scheme of network motifs with one and three mediated activators. The left (**A**) motif represents the coherent FFL of type 1, as classified elsewhere [46], and the right (**B**) one is its modified version. In the context of our study, Z_i_ stands for target genes, while X, Y, and Y_i_ are transcription and mobile factors.

In the first part of the present study, we theoretically analyzed a core network motif shown in Figure 2. We investigated a possible biological benefit that the presence of several regulatory branches in the motif may provide in the target regulation. In the second part, we considered four models with different combinations of *FTa1*, *SOC1a*, *SOC1b*, and *SOC1c* in the regulation of *PIM* in *M. trancatula*. The model solutions were fit to the gene expression data with and without vernalization in wild-type and *fta1-1* mutants. The weights of different regulation branches of the network in other legumes may differ from those in *M. trancatula*. Our approach allows the model to be extended by adding new branches and hubs as the new experimental data become available.

## 2. Materials and Methods

### 2.1. Mathematical Model for the Feed-Forward Loop of PIM Activation

*PIM* activation by an FT homolog (FTa1) directly and via three intermediate activators (SOC1a, SOC1b, and SOC1c) represents a variation of a feed-forward loop (FFL), which is simple but very widespread in biological networks [47]. It consists of one target Z that is activated by X both directly and indirectly via Y (Figure 2A). A biological function of this FFL is well characterized for various assumptions about possible ways to combine regulatory interactions and regulatory parameters, and this function can be described as a delayed response in target activity to a variation of X [46]. The appearance of several intermediate activators in this FFL, which is characteristic of legumes, leads to the general problem of studying this model network motif and comparing the modified loop with the simple one (Figure 2B).

For simplicity, we assume that indirect branches act independently, and there is no competition for the target between them. Admitting no essential influence of Y_i_ on the activation of Z_i_ by X, we can use kinetic equations with “OR logic” in combining all regulations, as described elsewhere [46]. In this case, the model describing the FFL with three intermediate activators is as follows:

(1) $$\begin{array}{c} \dot{Y_{i}}=\frac{a_{yi}X}{K_{yi}+X}-b_{y}Y_{i},\\ \dot{Z}=\frac{a_{xz}X}{K_{xz}+X}+\sum_{i}\frac{a_{yzi}Y_{i}}{K_{yzi}+Y_{i}}-b_{z}Z, \end{array}$$

where $a_{i},\;a_{xz},\;a_{yzi}$ are the maximal synthesis rate; $b_{y},\;b_{z}$ are degradation coefficients; the production rates are represented by the Hill functions with the dissociation coefficients $K_{yi},\;K_{xz},\;K_{yzi}$. All parameter values were set equal to 1 in this analysis. We assume no cooperative effects in Z regulation. The summation in Equation (1) occurs over the indirect branches in the FFL. Such systems of differential equations have unique solutions [48].

### 2.2. Mathematical Modeling of the Medicago trancatula Data

To obtain numerical data from the plots published in Ref. [33], we used WebPlotDigitizer software (https://automeris.io/WebPlotDigitizer Version 4.6, accessed on 4 December 2023). This web-based tool demonstrated high validity and reliability in extraction of digital data from a variety of plots [49].

**Figure 3:**
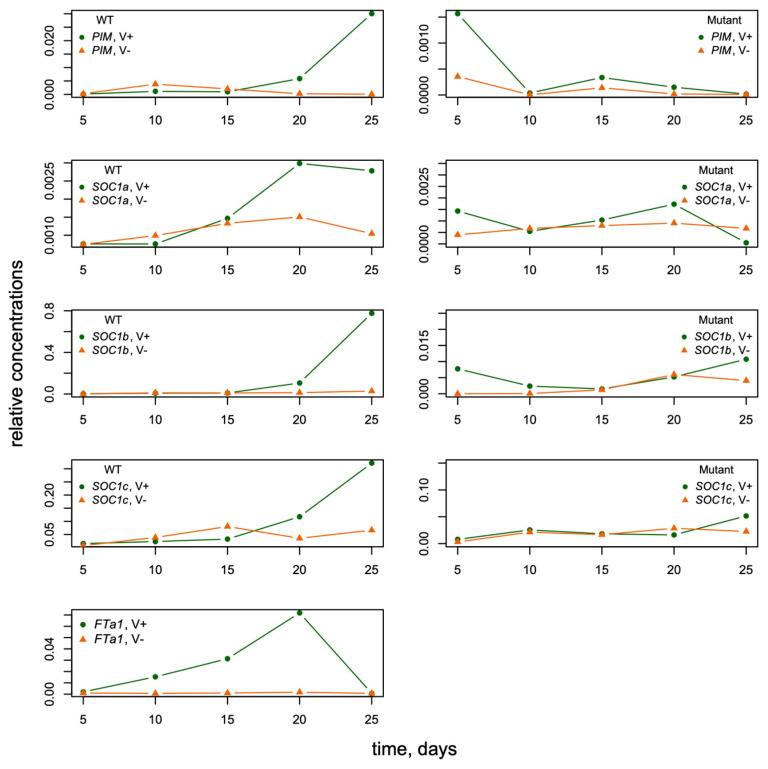
The dynamics of *FTa1* expression in leaves and *SOC1a*, *SOC1b*, *SOC1c*, and *PIM* expression in the shoot apexes of *M. trancatula* obtained by qRT-PCR (modified from ([33], Figure 4)). The dynamics of mean expression levels are shown for wild-type and *fta1-1* mutant plants with and without vernalization (“V+“ and “V−,“ respectively) and LD photoperiod. The data are normalized to the *PDF2* reference gene expression [33].

The data represented the expression dynamics of genes *PIM*, *FTa1*, *SOC1a*, *SOC1b*, and *SOC1c* during *M. trancatula* development (5–25 days after sowing) with and without vernalization and under the long-day (LD) growing conditions (Figure 3). All gene expression time courses were measured for wild-type and *fta1-1* mutant genotypes. Therefore, the data included mRNA concentrations (mean ± s.d.) of five genes at five time points in four cases (with/without vernalization, wild/mutant genotype). *FTa1* expression was significantly activated by vernalization and LD photoperiod in the leaves. It reached maximum by day 20 and declined thereafter. The expression levels of *SOC1a*, *SOC1b*, *SOC1c*, and *PIM* in the shoot apex also demonstrated a substantial induction by vernalization; however, this induction was observed later than for *FTa1*. The expression levels of these genes in *fta1-1* late flowering mutants were much lower compared to wild type, and no activation by vernalization was detected in the considered time interval [33] (Figure 3).

We elaborated dynamical models to fit these data, which were based on the kinetic equations with the Michaelis–Menten kinetics. Due to the limited amount of data, we assumed no cooperative effects in regulation to reduce the number of free parameters in the model equations.

We used mathematical modeling to test four hypotheses (H_1_–H_4_) about how the *FT* and *SOC1* genes combined in the activation of *PIM*. Different possible types of this combination were associated with different mathematical representations of the model equations, as described further separately for each hypothesis.

The H_1_ hypothesis represents the model where all activators regulate *PIM*, and the SOC1 transcription factors are functionally identical in terms of this regulation. The latter means that the contribution of the SOC1 activators can be presented via the sum of their concentration in the *PIM* synthesis rate, with the same kinetic parameters for all *SOC1*-like genes. The model for the *PIM* dynamics then has the following form:

(2) $$\frac{dPIM}{dt}=\nu_{0}\frac{FTa1(t-\tau )}{K_{0}+FTa1(t-\tau )}+\nu_{1}\frac{\left(SO{C1}_{a}(t)+SO{C1}_{b}(t)+SO{C1}_{c}(t)\right)}{K_{1}+(SO{C1}_{a}(t)+SO{C1}_{b}(t)+SO{C1}_{c}(t))}-\lambda PIM(t),$$

where PIM and SOC1_i_ are protein concentrations coded by *PIM* and the *SOC1* genes, respectively, in the apical meristem, FTa1 is the protein concentration coded by *FTa1* in leaves, $v_{i}$ are the maximal synthesis rates, λ is the degradation constant for PIM, and $K_{i}$ are the Michaelis constants (dissociation constants for the interaction between transcription factors and *PIM*’s regulatory region). We assume that it takes τ days to transport the FTa1 protein to the apical meristem, so we represent the FTa1 concentration in the apex at time *t* as FTa1(*t* − τ) in the model equations.

The H_2_ hypothesis suggests that the sum of the SOC1 proteins fully determines the *PIM* dynamics, and FTa1 does not participate in the direct *PIM* activation. The model for the *PIM* dynamics in this hypothesis takes the following form:

(3) $$\frac{dPIM}{dt}=\nu_{1}\frac{\left(SO{C1}_{a}(t)+SO{C1}_{b}(t)+SO{C1}_{c}(t)\right)}{K_{1}+(SO{C1}_{a}(t)+SO{C1}_{b}(t)+SO{C1}_{c}(t))}-\lambda PIM(t).$$

The H_3_ hypothesis again suggests that FTa1 has no sufficient effect on the *PIM* dynamics but additionally assumes that the SOC1 proteins are functionally different in the *PIM* activation. This means that each SOC1_i_(t) is associated with its own regulatory parameters in the equation for *PIM*. The model in this hypothesis has the following form:

(4) $$\frac{dPIM}{dt}=\nu_{1}\frac{SO{C1}_{a}(t)}{K_{1}+SO{C1}_{a}(t)}+\nu_{2}\frac{SO{C1}_{b}(t)}{K_{2}+SO{C1}_{b}(t)}+\nu_{3}\frac{SO{C1}_{c}(t)}{K_{3}+SO{C1}_{c}(t)}-\lambda PIM(t),$$

where the kinetic parameters *v_i_* and *K_i_* are different for different SOC1 proteins.

H_4_ assumes that all regulators have different contributions in the *PIM* activation and, hence, different kinetic parameters, so the model takes the following form:

(5) $$\frac{dPIM}{dt}=\nu_{0}\frac{FTa1(t-\tau )}{K_{0}+FTa1(t-\tau )}+\nu_{1}\frac{SO{C1}_{a}(t)}{K_{1}+SO{C1}_{a}(t)}+\nu_{2}\frac{SO{C1}_{b}(t)}{K_{2}+SO{C1}_{b}(t)}+\nu_{3}\frac{SO{C1}_{c}(t)}{K_{3}+SO{C1}_{c}(t)}-\lambda PIM(t).$$

Since *fta1* mutants still showed delayed flowering in the vernalized LD conditions, we did not consider fifth hypothesis that only FTa1 is involved in *PIM* regulation.

We find values of all parameters (maximal synthesis rates, degradation constants, dissociation constants, and time delay τ) by fitting models H_1_–H_4_ to the *Medicago* data, minimizing the following cost function *V*:

(6) $$V=\sum_{i=2}^{5}\left(\frac{{\left(Y_{i}^{+}-y_{i}^{+}\right)}^{2}}{\sigma_{1i}^{2}}+\frac{{\left(Y_{i}^{-}-y_{i}^{-}\right)}^{2}}{\sigma_{2i}^{2}}+\frac{{\left(Y_{i}^{mut+}-y_{i}^{mut+}\right)}^{2}}{\sigma_{3i}^{2}}+\frac{{\left(Y_{i}^{mut-}-y_{i}^{mut-}\right)}^{2}}{\sigma_{4i}^{2}}\right),$$

where *i* is the number of time point, *Y_i_* and *y_i_* represent average data values and model solutions, respectively, ‘+/−’ in superscripts, mark the presence or absence of vernalization, the presence/absence of ‘*mut*’ in superscripts denotes the mutant/wild type genotype, respectively, and σ’s are the data values of the standard deviations for data values at *i*th time point. We use mRNA concentrations from the data in Equation (6), assuming that protein concentrations are proportional to mRNA concentrations for simplicity. The summation in Equation (6) starts from *i* = 2 since data from the first time point are used as the initial conditions in the models. We ran the numerical minimization process 1000 times from different initial points using simulated anneal algorithm in MATLAB and selected the best result.

To choose between hypotheses, we used the Akaike information criterion (*AIC*) adjusted for small data samples to compare the models:

(7) $$AIC=2k-2log\hat{L}+\frac{2k^{2}+2k}{m-k-1},$$

where *k* is the number of optimized parameters, *m* is the number of data points, $2log\hat{L}=-V_{min}$ is the maximal value of the log-likelihood function expressed in terms of the minimal value of the minimized cost function [44]. *AIC* takes into account both the proximity of the model solution to data and the number of free parameters, as more sophisticated hypotheses possess larger numbers of free parameters and, thus, are prone to overfitting. More preferable hypotheses correspond to smaller *AIC* values.

## 3. Results

### 3.1. Multiple SOC1 Genes in the FFL of PIM Activation Ensure Buffering of FT Variation

To study the features of the general model, we compared models of two simple feed-forward loops shown in Figure 2. The first loop consisted of regulator X, which activated target Z_1_ directly and indirectly through the activation of regulator Y. In the second loop, in addition to the direct activation of target Z_2_ by X, there existed three intermediate branches with activators Y_1_, Y_2_, and Y_3_. These FFLs were modeled using simple mathematical models with Equation (1). Here, we had no interest in absolute concentration values and considered relative values. In the motif with three intermediate regulation branches, we suggested that all three regulators were similar, with the same kinetic parameters.

**Figure 4:**
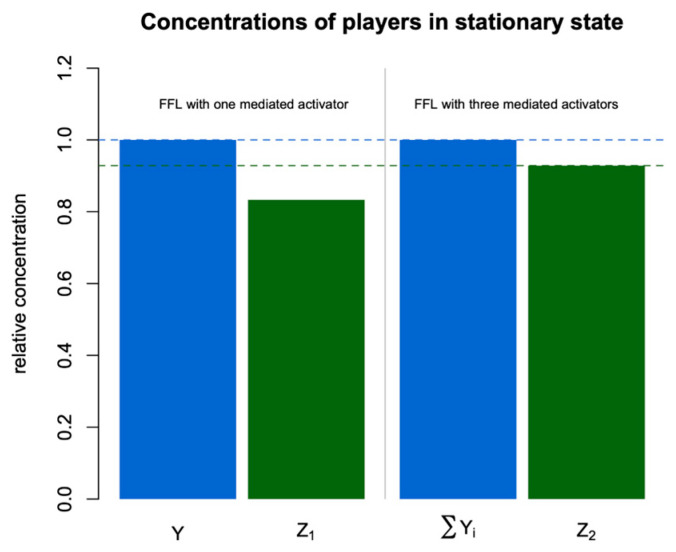
Stationary values of all concentrations relative to Y in the FFLs from Figure 2.

Figure 4 shows the relative concentrations of all players in the stationary state in the two regulatory motifs. As one can see, the total concentration of regulators is the same for both FFLs, but the target concentration is higher for the second FFL. Three independent branches in this regulatory motif provide more effective activation of the target, and this effect is most noticeable if these branches have approximately the same impact on regulation.

**Figure 5:**
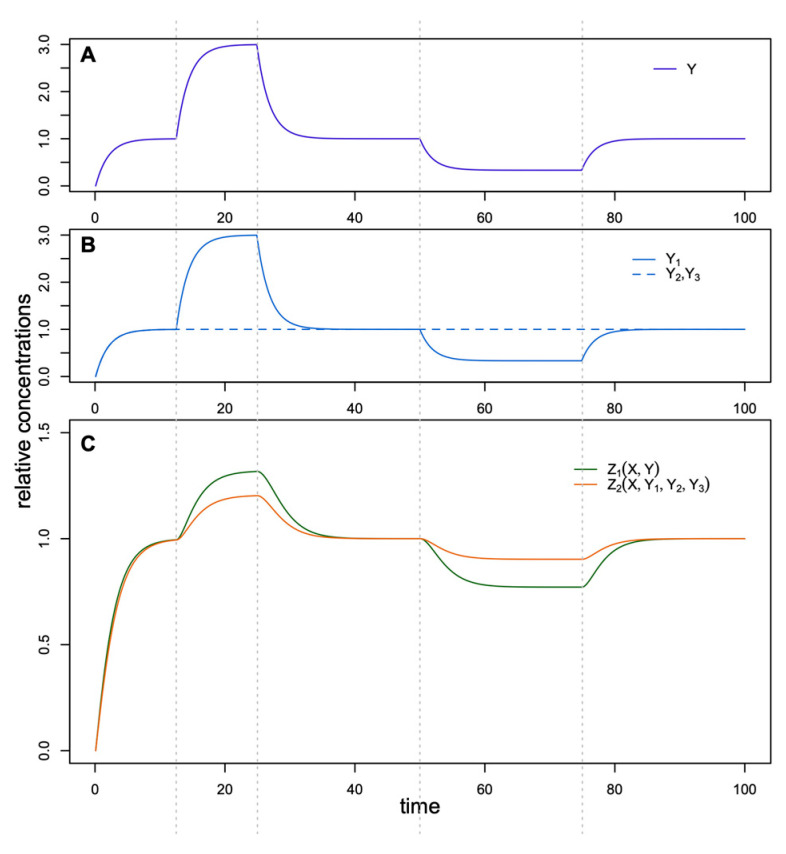
Dynamics of Z_1_ and Z_2_ (**C**) in the FFLs in response to variation of (**A**) Y and (**B**) Y_1_–Y_3_, respectively.

Another evident advantage of three independent intermediate regulators instead of one is buffering against possible deleterious mutations in some Y_i_ so that other Y_i_ can still fulfill regulation. Figure 5 demonstrates how changes in the intermediate regulators affect the target concentration. For the second FFL, a threefold variation of one regulator, Y_1_, leads to a smaller variation of the target Z_2_ as compared to the target Z_1_ and a similar variation of Y in the first FFL. To force noticeable changes in Z_2_, we need to activate or suppress all three intermediate activators simultaneously, which we can do by perturbing the upstream regulator X. In other words, the target in the FFL with three intermediate regulators essentially responds only to a ‘real signal’ that has the form of the perturbed upstream regulator X and filters variational signals from any single intermediate regulator Y_i_.

### 3.2. Modeling PIM Dynamics in M. trancatula Suggests Cumulative Activation by SOC1 Genes

We elaborated four mathematical models of *PIM* regulation by FTa1 and SOC1 proteins and applied them to the *M. trancatula* data to test the following hypotheses H_1_–H_4_ about how activators of *PIM* combine to determine its expression dynamics observed in the data (see Section 2 for more details):

- H_1_: *PIM* is regulated by both FTa1 and all SOC1 transcription factors, and all SOC1 transcription factors are not functionally distinguishable in this regulation.
- H_2_: *PIM* is regulated only by the SOC1 transcription factors, and all SOC1 transcription factors are not functionally distinguishable.
- H_3_: *PIM* is regulated only by the SOC1 transcription factors, and each SOC1 transcription factor has its own regulatory parameters.
- H_4_: *PIM* is regulated by both FTa1 and all SOC1 transcription factors, and each SOC1 transcription factor has its own regulatory parameters.

**Figure 6:**
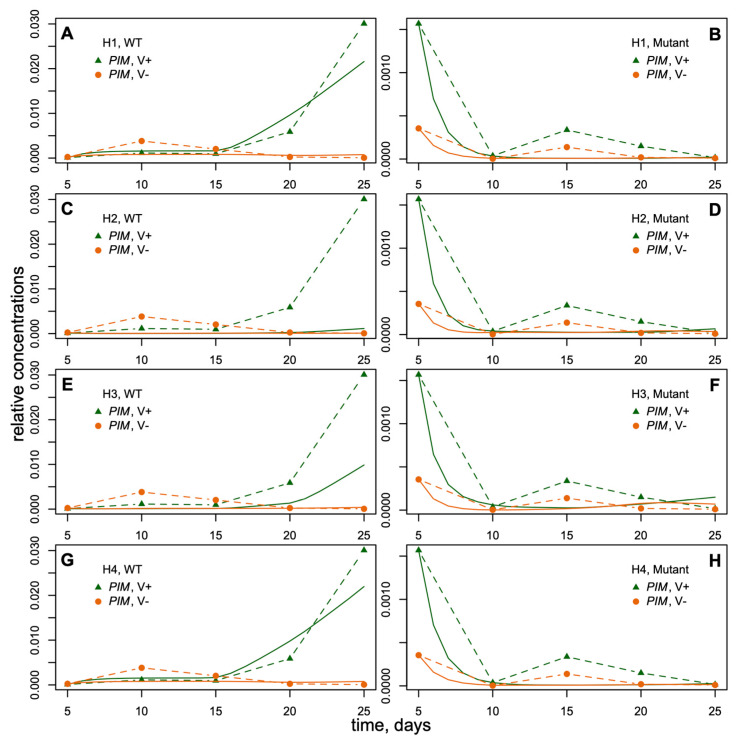
Dynamics of *PIM* expression in models (solid curves) and data (dashed curves). (**A**,**B**): model under H_1_; (**C**,**D**): model H_2_; (**E**,**F**): model H_3_; (**G**,**H**): model H_4_. V+, vernalization; V−, absence of vernalization; WT, wild type; mutant, *fta1-1* mutant plants.

We fitted the models H_1_–H_4_ to the expression data, minimizing the functional (6) for two genotypes (wild type and *fta1-1*) and in the presence or absence of vernalization. Table 1 shows the best parameter values for each model. The results demonstrated different fitting quality for different hypotheses (Figure 6). The *PIM* dynamics for the mutant genotype were visually similar in all models, so the main difference appeared for the wild type. The wild-type *PIM* expression in models H_1_ and H_4_ was close to data under vernalization (Figure 6A,G), while models H_2_ and H_3_ exhibited *PIM* underexpression (Figure 6C,E). This result hinted at the fact that the SOC1 transcription factors were not enough for *PIM* activation and that the direct activation from FTa1 was necessary since only models H_1_ and H_4_ implemented the direct *PIM* activation by FTa1. Prescribing separate regulatory parameters for each SOC1 in model H_3_ increased *PIM* expression as compared to the cumulative activation from SOC1a to SOC1c (Figure 6E vs. Figure 6C) but was not sufficient.

**Figure 7:**
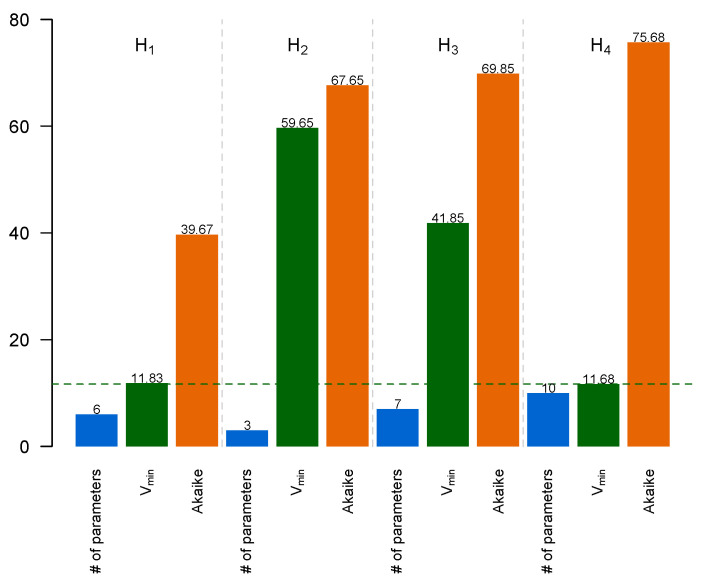
Number of free parameters in a model (blue), minimal cost function value *V*_min_ (green), and *AIC* value (brown) for models H_1_–H_4_.

To decide which model is preferable, we calculated the Akaike information criterion for each model, which accounted for both the minimal value of the minimized cost function and the number of free parameters in the model (Figure 7; see Section 2 for more details). Model H_4_ showed the smallest value, *V*_min_, of the cost function among all models, but this value did not noticeably differ from *V*_min_ in model H_1_. On the other hand, model H_4_ had 10 free parameters vs. 6 free parameters in H_1_. This balance between the number of degrees of freedom in the model and its ability to approximate data resulted in model H_1_ exhibiting the smallest *AIC*. Therefore, hypothesis H_1_ should be considered the most preferred among the tested hypotheses.

## 4. Discussion

### 4.1. Multiple SOC1 Genes Buffer FT Variation in the FFL of PIM Activation

The regulatory motif controlling the vernalization-induced flowering in legumes includes several activating branches. The number of controlling ways seems to be abundant, but studying even the simplest model uncovers the sense of these ways in such a significant process. Our theoretical analysis demonstrates that several intermediate activators in the flowering activation motif provide more effective activation of the target, and gene breakdown would not lead to vital changes. However, several activating branches force noticeable changes in the target, only working together simultaneously. This is possible in a case of upstream regulation, and variations in only one branch that are not supported by variations in others are smoothed away by the loop. In other words, the loop with several branches eliminates no-sense signals and amplifies the useful signal from an upstream regulator. Since flowering is one of the most substantial processes of the plant, the support of its activation needs to be very reliable.

### 4.2. The Cumulative PIM Activation by Three SOC1 Genes Is Favored in the M. trancatula Model

The published experimental results showed that all three *M. trancatula SOC1* genes promoted the early flowering of the *Arabidopsis soc1–2* mutant, which points to their functional identity with the *Arabidopsis SOC1* [33]. Nevertheless, there were noticeable differences in both the levels and dynamics of *M. trancatula SOC1s* expression upon induction by vernalization (Figure 3). Moreover, *SOC1s* had different effects on flowering time. The flowering was delayed significantly in the *M. trancatula SOC1a* mutants, while the mutation in the *SOC1b* gene did not affect flowering time. An input of the *SOC1c* gene has not yet been clarified [33,38].

These differences in *SOC1s* functions are reflected in the fits of H_2_ and H_3_ models to experimental data on *PIM* expression (Figure 6C,E). In the absence of FTa1, neither of the two models show good fits, but the differences between the independent regulation by different SOC1s (H_3_) and their cumulative input (H_2_) are quite evident. However, these differences become indistinguishable in the models, including *FTa1*, the major integrator of the vernalization and LD signals. Thus, in the real plant, the direct regulation of the target by FT is more intensive than regulation from intermediate branches of the motif (Figure 2). Indeed, the model including both FTa1 and SOC1s, where SOC1s act in sum (H_1_), is no worse than the complex model with SOC1s acting independently (H_4_) (Figure 6A,G). Moreover, the *AIC* value is much lower in the H_1_ model compared to H_4_ model (Figure 7). This led to a conclusion that, in the *M. trancatula* models, it makes no sense to consider different *SOC1* genes as independent activators with specific regulatory properties. This is in line with the suggested hypothesis of functional redundancy of *SOC1* genes [33,38].

Moreover, modeling the *M. trancatula* data under different hypotheses resulted in a curious trend: values of the SOC1-related dissociation constants *K*_1_, *K*_2_, and *K*_3_ were much higher than the FTa1-related dissociation constant *K*_0_. The results were similar even if FTa1 was absent in the model (Table 1). This result suggests a small impact of the intermediate activators.

In *Arabidopsis*, an induction of *SOC1* by vernalization is both dependent and independent of the *FT* gene [15]. In legumes, a possibility of the *FTa1*-independent *SOC1* regulation still exists since the late flowering of the *fta1* mutants is detected under vernalization and LD [38]. The only known floral repressor, whose mutation enables bypassing the vernalization requirement in legumes, is the *M. trancatula* ortholog of the *Arabidopsis VRN2* gene. The mutation in *VRN2* resulted in the increased expression of *FTa1* and all three *SOC1* genes under LD [50]. However, no detailed analyses of the direct targets or interaction partners of *VRN2* have been conducted. *SOC1s* may also be targeted by the LD-induced *FT* genes, e.g., the *FTb1* gene [28]. Nevertheless, a possible independent input of *SOC1* genes in the vernalization-induced flowering cannot be neglected.

*PIM* expression in *M. trancatula fta1-1* mutants starts to increase with a delay compared with the wild type [33,38]. Within the considered period of 25 days, this concentration is very small (Figure 3 and Figure 6). Interestingly, all four models fit the mutant data in a similar way (Figure 6B,D,F,H), indicating that the *PIM* expression in this time interval is independent of structural changes in the regulatory module.

The network motif considered in this study can be supplemented by additional regulatory nodes as new experimental data become available. The weights of the regulation branches in other legumes may differ from those in *M. trancatula*, and modeling data from other crops will elucidate regulation processes in the future. Therefore, our study can be considered as a first step towards the quantitative understanding of regulatory mechanisms induced by vernalization.

### 4.3. Limitations of the Modeling Approach

The modeling of complex biological processes is always challenging and can be achieved through a number of simplifications. Such simplifications are largely determined by the limited resolution of experimental data. At the molecular level, the data on gene expression often lack information on absolute concentrations, half-lives, or mRNA/protein binding affinities. Moreover, the mathematical models often operate with large network hubs, which are further complemented by a more complete set of regulators.

The dynamic modeling of gene expression requires data that changes over time or space. For vernalization in legumes, a dataset that meets these requirements is only available for *Medicago trancatula* and includes temporal dynamics of flowering gene expression during vernalization at the RNA level [33]. Due to the lack of experimental data on protein expression, in the model, we assumed that protein concentrations are proportional to corresponding mRNA concentrations. As a consequence, this proportionality coefficient appears as a multiplier factor in the values of the equilibrium dissociation constants *K*, which represent the interaction of the regulator proteins with the target gene promoters. We find the values of *K* by fitting the model to experimental data (Table 1), so we cannot extract potentially interesting information about DNA–protein interaction from these values or use any experimental estimates on the dissociation constants to prescribe *K* in the model.

Our approach also has limitations at the gene network level. As shown in *Arabidopsis*, the integrator genes *FT* and *SOC1* summarize information from the upstream pathways and transfer it to downstream meristem identity genes to promote flowering [5,6] (Figure 1A). The activation role of these integrators is proposed to be conserved in legumes [28,31,33,34]. The expression data for *M. trancatula* are available for the *FTa1* and three *SOC1* genes (Figure 1B); however, there is very limited information on candidate genes acting upstream of these integrators. A simplification of the model is that we do not explicitly consider information on the regulatory inputs of the upstream pathways but assume that this information is pre-included in the expression of pathway integrator genes (“hubs”). The earlier in silico study has shown that flowering time depends on the expression levels of integrator genes [51]; thus, this simplification is quite reliable. This way of generalizing the structure of gene networks has previously been used in numerous models of floral transition in *Arabidopsis* [40,41,43] and legumes [44,45]. Using a “hub” regulatory module enabled us to successfully fit the model to the data and test four hypotheses of *PIM* regulation in *M. trancatula*. These hypotheses can be further verified in experiments, and the newly obtained experimental data, in turn, can be included in the model.

The values of free parameters in the model were obtained by optimization, so the risk of overfitting exists. To reduce this potential risk, we used the systematic ensemble approach for the analysis of the optimization results [52]. All our conclusions were based on the average values over the ensemble of optimized parameter values.

Despite the limitations, in this study, we applied rigorous mathematical methods that allowed us to test various hypotheses using available experimental data. The presented modeling approach can be further applied to build more complete models once detailed gene expression and interaction data become available.

## Figures and Tables

**Table 1:** Best parameter values in models H_1_–H_4_.

Parameter	H_1_	H_2_	H_3_	H_4_
Max synthesis rate provided by FTa1 (*v*_0_)	0.98	–	–	0.86
FTa1–*PIM* dissociation constant (*K*_0_)	1.55	–	–	1.41
PIM degradation rate (*λ*)	0.82	1.00	1.00	0.81
FTa1-related time delay (*τ*_0_)	9.90	–	–	9.67
Max synthesis rate provided by ΣSOC1i (*ν_i_*)	0.34	1.00	–	–
ΣSOC1i–*PIM* dissociation rate (*K*_1_)	996	814	–	–
Max synthesis rate provided by SOC1a (*ν*_1_)	–	–	0.008	0.97
SOC1a–*PIM* dissociation rate (*K*_1_)	–	–	979	997
Max synthesis rate provided by SOC1b (*ν*_2_)	–	–	0.99	0.97
SOC1b–*PIM* dissociation rate (*K*_2_)	–	–	63.9	945
Max synthesis rate provided by SOC1c (*ν*_3_)	–	–	0.0003	0.27
SOC1c–*PIM* dissociation rate (*K*_3_)	–	–	931	983

## Data Availability

All data and modeling results are contained within the article.
